# 5-(4-Methyl­phen­yl)-3-phenyl­cyclo­hex-2-en-1-one

**DOI:** 10.1107/S1600536812027031

**Published:** 2012-06-20

**Authors:** Shaaban K. Mohamed, Mehmet Akkurt, Antar A Abdelhamid, Kuldip Singh, Omyma A. A. Abd Allah

**Affiliations:** a Chemistry and Environmental Division, Manchester Metropolitan University, Manchester M1 5GD, England; b Department of Physics, Faculty of Sciences, Erciyes University, 38039 Kayseri, Turkey; c Department of Chemistry, University of Leicester, Leicester, England; d Department of Chemistry, Faculty of Science, Sohag University, Sohag, Egypt

## Abstract

In the title compound, C_19_H_18_O, the cyclo­hexene ring has an envelope conformation with the methine C atom on the flap. The phenyl and methyl­phenyl rings form a dihedral angle of 85.93 (11)°. The crystal packing is consolidated by van der Waals forces and weak C—H⋯π inter­actions.

## Related literature

For the biological activity of α,β-unsaturated carbonyl compounds, see: Podraze (1991 ▶); Suksamrarn *et al.* (2003 ▶); Modzelewska *et al.* (2006 ▶); Shettigar *et al.* (2006 ▶); Ferrer *et al.* (2009 ▶); Asiri (2003 ▶); Forestier *et al.* (1989 ▶); Kumar *et al.* (2003 ▶). For the synthesis of cyclo­hexenones, see: Diao & Stahl (2011 ▶); González *et al.* (2009 ▶); Zhang *et al.* (2008 ▶). For geometric analysis, see: Cremer & Pople (1975 ▶).
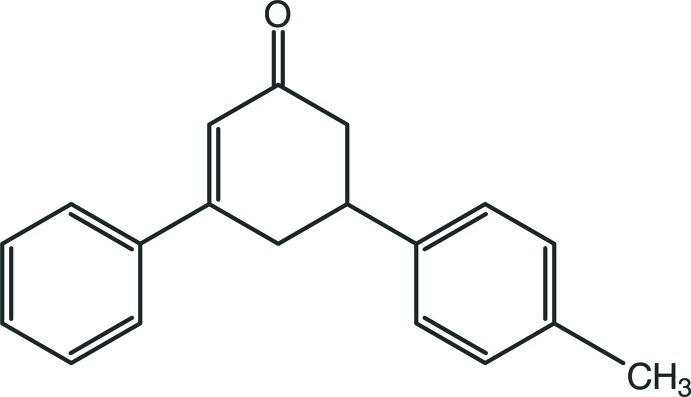


## Experimental

### 

#### Crystal data

C_19_H_18_O
*M* *_r_* = 262.33
Monoclinic, 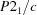

*a* = 17.085 (4) Å
*b* = 5.6807 (11) Å
*c* = 15.689 (3) Å
β = 113.152 (4)°
*V* = 1400.1 (5) Å^3^
*Z* = 4
Mo *K*α radiation
μ = 0.08 mm^−1^
*T* = 150 K
0.42 × 0.24 × 0.12 mm

#### Data collection

Bruker APEX 2000 CCD area-detector diffractometer
Absorption correction: multi-scan (*SADABS*; Bruker, 2005 ▶) *T*_min_ = 0.979, *T*_max_ = 0.991
9636 measured reflections
2473 independent reflections
1497 reflections with *I* > 2σ(*I*)
*R*_int_ = 0.106

#### Refinement

*R*[*F* ^2^ > 2σ(*F* ^2^)] = 0.053
*wR*(*F* ^2^) = 0.114
*S* = 0.90
2473 reflections
182 parameters
H-atom parameters constrained
Δρ_max_ = 0.22 e Å^−3^
Δρ_min_ = −0.19 e Å^−3^

### 

Data collection: *SMART* (Bruker, 2005 ▶); cell refinement: *SAINT* (Bruker, 2005 ▶); data reduction: *SAINT*; program(s) used to solve structure: *SHELXS97* (Sheldrick, 2008 ▶); program(s) used to refine structure: *SHELXL97* (Sheldrick, 2008 ▶); molecular graphics: *ORTEP-3 for Windows* (Farrugia, 1997 ▶) and *PLATON* (Spek, 2009 ▶); software used to prepare material for publication: *WinGX* (Farrugia, 1999 ▶) and *PLATON*.

## Supplementary Material

Crystal structure: contains datablock(s) global, I. DOI: 10.1107/S1600536812027031/xu5565sup1.cif

Structure factors: contains datablock(s) I. DOI: 10.1107/S1600536812027031/xu5565Isup2.hkl

Supplementary material file. DOI: 10.1107/S1600536812027031/xu5565Isup3.cml

Additional supplementary materials:  crystallographic information; 3D view; checkCIF report

## Figures and Tables

**Table 1:** Hydrogen-bond geometry (Å, °) *Cg* is the centroid of the C13–C18 benzene ring.

*D*—H⋯*A*	*D*—H	H⋯*A*	*D*⋯*A*	*D*—H⋯*A*
C10—H10⋯*Cg* ^i^	0.95	2.77	3.601 (3)	147

Symmetry code: (i) 
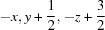
.

**Fig. 1.**
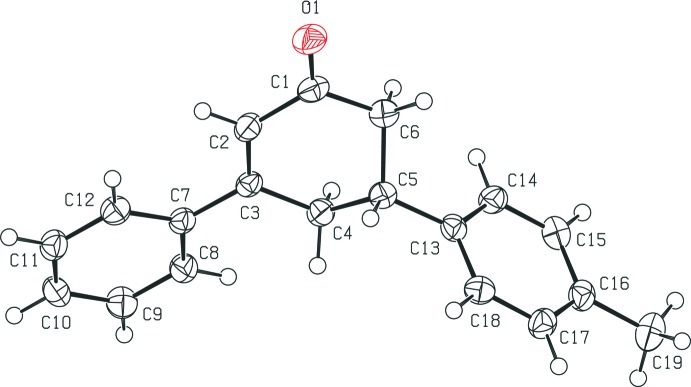
The molecular structure of the title compound with the atom numbering scheme. Displacement ellipsoids for non-H atoms are drawn at the 50% probability level.

**Fig. 2.**
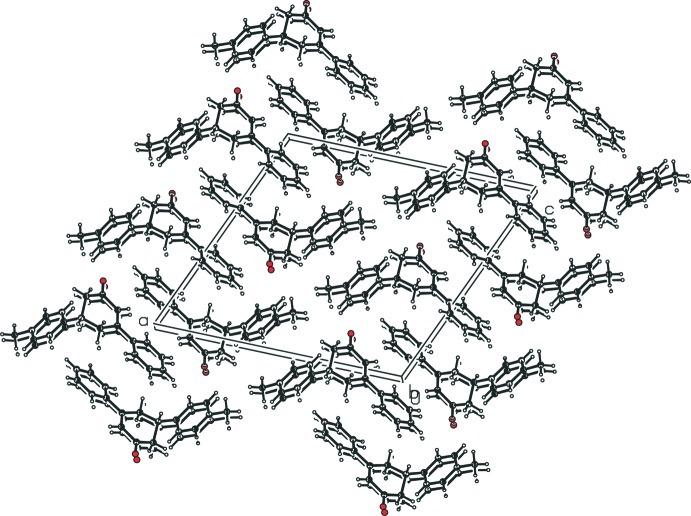
View of the molecular packing of the title compound along the b axis.

## supplementary crystallographic information

### Comment

α,β-Unsaturated carbonyl compounds have shown various biological activities such as antioxidant (Suksamrarn *et al.*, 2003), antitumor (Kumar *et al*., 2003), anticancer (Modzelewska *et al.*, 2006) and antimalarial (Ferrer *et al.*, 2009). In addition, chalcones also were widely used in cosmetic compositions (Forestier *et al.*, 1989; Podraze, 1991) and applications of dyes (Asiri, 2003). Apart from being biologically important compounds, chalcone derivatives show non-linear optical (NLO) properties with excellent blue light transmittance and good crystallizability (Shettigar *et al*., 2006). In this context, herein we report the synthesis and crystal structure of the title compound (I).

As seen in Fig. 1, the title compound is not planar. The C1–C6 cyclohexene ring in (I) has a nearly envelope conformation [puckering parameters (Cremer & Pople, 1975) Q_T_ = 0.511 (3) Å, θ = 53.4 (3) ° and φ = 247.6 (4) °]. The C7–C12 phenyl ring makes a dihedral angle of 85.93 (11)° with the methyl-substituted C13 C18 benzene ring.

The crystal packing of (I) is stabilized by van der Waals forces and weak C—H···π interactions (Table 1, Fig. 2).

### Experimental

To a solution of 222 mg (1 mmol) (2E)-3-(4-methylphenyl)-1-phenylprop-2-en-1-one in 40 ml e thanol, 100 mg of acetyl acetone was added in presence of 60 mg MeONa. The reaction mixture was refluxed for 7 h then cooled to room temperature (Diao & Stahl, 2011; González *et al.*, 2009; Zhang *et al.*, 2008). The excess solvent was removed under vacuum to afford the solid product which was filltered off and recrystallized from ethanol. The obtained crystals were in good quality (m.p. 343 K) for X-ray diffraction without further crystallization.

### Refinement

All H atoms were positioned geometrically (C—H = 0.95–1.00 Å) and refined by using a riding model, and with *U*_iso_(H) = 1.2 (1.5 for methyl groups)*U*_eq_(C).

### Crystal data

**Table:**

C_19_H_18_O	*F*(000) = 560
*M**_r_* = 262.33	*D*_x_ = 1.245 Mg m^−^^3^
Monoclinic, *P*2_1_/*c*	Mo *K*α radiation, λ = 0.71073 Å
Hall symbol: -P 2ybc	Cell parameters from 879 reflections
*a* = 17.085 (4) Å	θ = 2.6–28.4°
*b* = 5.6807 (11) Å	µ = 0.08 mm^−^^1^
*c* = 15.689 (3) Å	*T* = 150 K
β = 113.152 (4)°	Plate, colourless
*V* = 1400.1 (5) Å^3^	0.42 × 0.24 × 0.12 mm
*Z* = 4	

### Data collection

**Table:**

Bruker APEX 2000 CCD area-detector diffractometer	2473 independent reflections
Radiation source: fine-focus sealed tube	1497 reflections with *I* > 2σ(*I*)
Graphite monochromator	*R*_int_ = 0.106
phi and ω scans	θ_max_ = 25.0°, θ_min_ = 2.6°
Absorption correction: multi-scan (*SADABS*; Bruker, 2005)	*h* = −20→20
*T*_min_ = 0.979, *T*_max_ = 0.991	*k* = −6→6
9636 measured reflections	*l* = −18→18

### Refinement

**Table:**

Refinement on *F*^2^	Primary atom site location: structure-invariant direct methods
Least-squares matrix: full	Secondary atom site location: difference Fourier map
*R*[*F*^2^ > 2σ(*F*^2^)] = 0.053	Hydrogen site location: inferred from neighbouring sites
*wR*(*F*^2^) = 0.114	H-atom parameters constrained
*S* = 0.90	*w* = 1/[σ^2^(*F*_o_^2^) + (0.040*P*)^2^] where *P* = (*F*_o_^2^ + 2*F*_c_^2^)/3
2473 reflections	(Δ/σ)_max_ < 0.001
182 parameters	Δρ_max_ = 0.22 e Å^−^^3^
0 restraints	Δρ_min_ = −0.19 e Å^−^^3^

### Special details

**Table:**

Geometry. Bond distances, angles *etc*. have been calculated using the rounded fractional coordinates. All su's are estimated from the variances of the (full) variance-covariance matrix. The cell e.s.d.'s are taken into account in the estimation of distances, angles and torsion angles
Refinement. Refinement on *F*^2^ for ALL reflections except those flagged by the user for potential systematic errors. Weighted *R*-factors *wR* and all goodnesses of fit *S* are based on *F*^2^, conventional *R*-factors *R* are based on *F*, with *F* set to zero for negative *F*^2^. The observed criterion of *F*^2^ > σ(*F*^2^) is used only for calculating -*R*-factor-obs *etc*. and is not relevant to the choice of reflections for refinement. *R*-factors based on *F*^2^ are statistically about twice as large as those based on *F*, and *R*-factors based on ALL data will be even larger.

### Fractional atomic coordinates and isotropic or equivalent isotropic displacement parameters (Å^2^)

**Table:**

	*x*	*y*	*z*	*U*_iso_*/*U*_eq_	
O1	0.26608 (10)	0.8925 (3)	1.12374 (11)	0.0414 (6)	
C1	0.23739 (15)	0.7390 (4)	1.06451 (16)	0.0310 (8)	
C2	0.14596 (15)	0.7212 (4)	1.00905 (15)	0.0297 (8)	
C3	0.11153 (14)	0.5617 (4)	0.94080 (15)	0.0240 (8)	
C4	0.16904 (13)	0.3960 (4)	0.91730 (15)	0.0279 (8)	
C5	0.25840 (14)	0.4974 (4)	0.94353 (15)	0.0278 (8)	
C6	0.29405 (14)	0.5614 (4)	1.04625 (15)	0.0329 (8)	
C7	0.01837 (14)	0.5429 (4)	0.88700 (15)	0.0253 (8)	
C8	−0.01655 (14)	0.3458 (4)	0.83212 (15)	0.0303 (8)	
C9	−0.10293 (15)	0.3268 (4)	0.78170 (16)	0.0337 (9)	
C10	−0.15772 (14)	0.5021 (4)	0.78364 (16)	0.0312 (8)	
C11	−0.12489 (15)	0.6993 (4)	0.83717 (16)	0.0315 (9)	
C12	−0.03831 (14)	0.7195 (4)	0.88790 (15)	0.0299 (8)	
C13	0.31421 (13)	0.3378 (4)	0.91407 (15)	0.0258 (8)	
C14	0.34825 (14)	0.1297 (4)	0.96021 (16)	0.0297 (8)	
C15	0.39595 (14)	−0.0172 (4)	0.92875 (16)	0.0326 (8)	
C16	0.41086 (14)	0.0364 (4)	0.85029 (16)	0.0298 (8)	
C17	0.37680 (14)	0.2433 (4)	0.80440 (17)	0.0315 (8)	
C18	0.32954 (14)	0.3917 (4)	0.83590 (16)	0.0299 (8)	
C19	0.46239 (15)	−0.1244 (5)	0.81565 (18)	0.0438 (10)	
H2	0.10900	0.82740	1.02180	0.0360*	
H4A	0.14380	0.36270	0.84990	0.0330*	
H4B	0.17310	0.24530	0.95050	0.0330*	
H5	0.25190	0.64790	0.90840	0.0330*	
H6A	0.29790	0.41820	1.08370	0.0390*	
H6B	0.35200	0.62730	1.06470	0.0390*	
H8	0.01990	0.22230	0.82950	0.0360*	
H9	−0.12500	0.19050	0.74500	0.0400*	
H10	−0.21720	0.48770	0.74870	0.0370*	
H11	−0.16200	0.82180	0.83910	0.0380*	
H12	−0.01680	0.85660	0.92430	0.0360*	
H14	0.33870	0.08750	1.01390	0.0360*	
H15	0.41900	−0.15800	0.96180	0.0390*	
H17	0.38590	0.28460	0.75030	0.0380*	
H18	0.30720	0.53340	0.80320	0.0360*	
H19A	0.42480	−0.19810	0.75740	0.0660*	
H19B	0.48940	−0.24660	0.86200	0.0660*	
H19C	0.50630	−0.03270	0.80500	0.0660*	

### Atomic displacement parameters (Å^2^)

**Table:**

	*U*^11^	*U*^22^	*U*^33^	*U*^12^	*U*^13^	*U*^23^
O1	0.0401 (11)	0.0482 (12)	0.0358 (10)	−0.0067 (9)	0.0149 (9)	−0.0171 (9)
C1	0.0361 (15)	0.0347 (15)	0.0259 (14)	−0.0045 (12)	0.0162 (12)	−0.0028 (12)
C2	0.0302 (14)	0.0327 (15)	0.0303 (14)	0.0009 (11)	0.0162 (12)	−0.0025 (12)
C3	0.0284 (14)	0.0235 (14)	0.0236 (12)	0.0014 (11)	0.0139 (11)	0.0030 (11)
C4	0.0288 (14)	0.0281 (14)	0.0275 (13)	0.0007 (11)	0.0119 (11)	−0.0002 (11)
C5	0.0288 (13)	0.0285 (14)	0.0260 (14)	0.0006 (11)	0.0107 (11)	−0.0017 (11)
C6	0.0318 (14)	0.0377 (16)	0.0287 (14)	0.0005 (12)	0.0113 (12)	−0.0038 (12)
C7	0.0275 (13)	0.0268 (14)	0.0242 (13)	0.0022 (11)	0.0129 (11)	0.0037 (11)
C8	0.0296 (14)	0.0297 (15)	0.0310 (14)	0.0024 (11)	0.0114 (11)	−0.0023 (12)
C9	0.0326 (15)	0.0304 (15)	0.0365 (15)	−0.0035 (12)	0.0119 (12)	−0.0050 (12)
C10	0.0238 (13)	0.0362 (16)	0.0303 (14)	0.0009 (11)	0.0071 (11)	0.0056 (12)
C11	0.0319 (15)	0.0313 (15)	0.0336 (15)	0.0065 (12)	0.0153 (12)	0.0033 (12)
C12	0.0324 (15)	0.0275 (14)	0.0305 (14)	−0.0014 (11)	0.0132 (12)	−0.0029 (11)
C13	0.0214 (13)	0.0275 (14)	0.0266 (13)	−0.0026 (11)	0.0073 (11)	−0.0027 (11)
C14	0.0293 (14)	0.0347 (15)	0.0274 (13)	−0.0023 (12)	0.0135 (11)	0.0008 (12)
C15	0.0276 (14)	0.0294 (15)	0.0384 (15)	0.0008 (11)	0.0105 (12)	0.0015 (12)
C16	0.0230 (13)	0.0312 (15)	0.0351 (14)	−0.0040 (11)	0.0113 (11)	−0.0062 (12)
C17	0.0282 (14)	0.0359 (15)	0.0338 (15)	−0.0041 (12)	0.0159 (12)	−0.0028 (12)
C18	0.0314 (14)	0.0285 (15)	0.0283 (13)	−0.0031 (11)	0.0103 (11)	−0.0012 (11)
C19	0.0377 (16)	0.0459 (17)	0.0542 (17)	0.0025 (13)	0.0250 (14)	−0.0032 (14)

### Geometric parameters (Å, º)

**Table:**

O1—C1	1.227 (3)	C16—C19	1.510 (4)
C1—C2	1.462 (4)	C17—C18	1.386 (3)
C1—C6	1.502 (4)	C2—H2	0.9500
C2—C3	1.348 (3)	C4—H4A	0.9900
C3—C4	1.507 (3)	C4—H4B	0.9900
C3—C7	1.484 (3)	C5—H5	1.0000
C4—C5	1.529 (3)	C6—H6A	0.9900
C5—C6	1.526 (3)	C6—H6B	0.9900
C5—C13	1.514 (3)	C8—H8	0.9500
C7—C8	1.395 (3)	C9—H9	0.9500
C7—C12	1.398 (3)	C10—H10	0.9500
C8—C9	1.378 (4)	C11—H11	0.9500
C9—C10	1.375 (4)	C12—H12	0.9500
C10—C11	1.380 (3)	C14—H14	0.9500
C11—C12	1.382 (4)	C15—H15	0.9500
C13—C14	1.389 (3)	C17—H17	0.9500
C13—C18	1.385 (3)	C18—H18	0.9500
C14—C15	1.386 (3)	C19—H19A	0.9800
C15—C16	1.385 (3)	C19—H19B	0.9800
C16—C17	1.382 (3)	C19—H19C	0.9800
			
O1—C1—C2	121.0 (2)	C5—C4—H4B	109.00
O1—C1—C6	121.8 (2)	H4A—C4—H4B	108.00
C2—C1—C6	117.3 (2)	C4—C5—H5	107.00
C1—C2—C3	123.3 (2)	C6—C5—H5	107.00
C2—C3—C4	119.4 (2)	C13—C5—H5	107.00
C2—C3—C7	122.4 (2)	C1—C6—H6A	110.00
C4—C3—C7	118.20 (19)	C1—C6—H6B	110.00
C3—C4—C5	112.14 (19)	C5—C6—H6A	110.00
C4—C5—C6	108.40 (19)	C5—C6—H6B	110.00
C4—C5—C13	111.98 (19)	H6A—C6—H6B	108.00
C6—C5—C13	115.3 (2)	C7—C8—H8	119.00
C1—C6—C5	110.00 (19)	C9—C8—H8	119.00
C3—C7—C8	120.8 (2)	C8—C9—H9	119.00
C3—C7—C12	122.3 (2)	C10—C9—H9	119.00
C8—C7—C12	116.9 (2)	C9—C10—H10	121.00
C7—C8—C9	121.3 (2)	C11—C10—H10	121.00
C8—C9—C10	121.0 (2)	C10—C11—H11	120.00
C9—C10—C11	118.9 (2)	C12—C11—H11	120.00
C10—C11—C12	120.3 (2)	C7—C12—H12	119.00
C7—C12—C11	121.6 (2)	C11—C12—H12	119.00
C5—C13—C14	122.4 (2)	C13—C14—H14	120.00
C5—C13—C18	119.9 (2)	C15—C14—H14	120.00
C14—C13—C18	117.6 (2)	C14—C15—H15	119.00
C13—C14—C15	120.8 (2)	C16—C15—H15	119.00
C14—C15—C16	121.6 (2)	C16—C17—H17	119.00
C15—C16—C17	117.6 (2)	C18—C17—H17	119.00
C15—C16—C19	121.6 (2)	C13—C18—H18	119.00
C17—C16—C19	120.8 (2)	C17—C18—H18	119.00
C16—C17—C18	121.1 (2)	C16—C19—H19A	109.00
C13—C18—C17	121.4 (2)	C16—C19—H19B	109.00
C1—C2—H2	118.00	C16—C19—H19C	109.00
C3—C2—H2	118.00	H19A—C19—H19B	110.00
C3—C4—H4A	109.00	H19A—C19—H19C	109.00
C3—C4—H4B	109.00	H19B—C19—H19C	110.00
C5—C4—H4A	109.00		
			
O1—C1—C2—C3	177.4 (2)	C6—C5—C13—C18	−132.9 (2)
C6—C1—C2—C3	−3.0 (3)	C3—C7—C8—C9	−179.8 (2)
O1—C1—C6—C5	−146.2 (2)	C12—C7—C8—C9	−0.3 (3)
C2—C1—C6—C5	34.2 (3)	C3—C7—C12—C11	179.8 (2)
C1—C2—C3—C4	−1.8 (3)	C8—C7—C12—C11	0.3 (3)
C1—C2—C3—C7	178.4 (2)	C7—C8—C9—C10	0.1 (4)
C2—C3—C4—C5	−25.3 (3)	C8—C9—C10—C11	0.0 (4)
C7—C3—C4—C5	154.5 (2)	C9—C10—C11—C12	0.0 (4)
C2—C3—C7—C8	−165.5 (2)	C10—C11—C12—C7	−0.2 (4)
C2—C3—C7—C12	15.0 (4)	C5—C13—C14—C15	177.1 (2)
C4—C3—C7—C8	14.8 (3)	C18—C13—C14—C15	0.2 (4)
C4—C3—C7—C12	−164.7 (2)	C5—C13—C18—C17	−176.7 (2)
C3—C4—C5—C6	55.5 (2)	C14—C13—C18—C17	0.3 (4)
C3—C4—C5—C13	−176.21 (18)	C13—C14—C15—C16	−0.5 (4)
C4—C5—C6—C1	−59.4 (2)	C14—C15—C16—C17	0.4 (4)
C13—C5—C6—C1	174.2 (2)	C14—C15—C16—C19	−179.6 (2)
C4—C5—C13—C14	−74.3 (3)	C15—C16—C17—C18	0.1 (4)
C4—C5—C13—C18	102.5 (2)	C19—C16—C17—C18	−179.9 (2)
C6—C5—C13—C14	50.3 (3)	C16—C17—C18—C13	−0.4 (4)

### Hydrogen-bond geometry (Å, º)

Cg is the centroid of the C13–C18 benzene ring.

**Table:**

*D*—H···*A*	*D*—H	H···*A*	*D*···*A*	*D*—H···*A*
C10—H10···*Cg*^i^	0.95	2.77	3.601 (3)	147

Symmetry code: (i) −*x*, *y*+1/2, −*z*+3/2.
